# Chondrosarcoma and Peroxisome Proliferator-Activated Receptor

**DOI:** 10.1155/2008/250568

**Published:** 2008-08-17

**Authors:** K. Nishida, T. Kunisada, Z. N. Shen, Y. Kadota, K. Hashizume, T. Ozaki

**Affiliations:** ^1^Department of Human Morphology, Graduate School of Medicine, Dentistry and Pharmaceutical Sciences, Okayama University, 2-5-1 Shikata-cho, Okayama 700-8558, Japan; ^2^Department of Orthopaedic Surgery, Graduate School of Medicine, Dentistry and Pharmaceutical Sciences, Okayama University, 2-5-1 Shikata-cho, Okayama 700-8558, Japan

## Abstract

Induction of differentiation and apoptosis in cancer cells by ligands of PPAR*γ* is a novel therapeutic approach to malignant tumors. Chondrosarcoma (malignant cartilage tumor) and OUMS-27 cells (cell line established from grade III human chondrosarcoma) express PPAR*γ*. PPAR*γ* ligands inhibited cell proliferation in a dose-dependent manner, and induced apoptosis of OUMS-27. The higher-grade chondrosarcoma expressed a higher amount of antiapoptotic Bcl-xL in vivo. The treatment of OUMS-27 by 15d-PGJ_2_, the most potent endogenous ligand for PPAR*γ*, downregulated expression of Bcl-xL and induced transient upregulation of proapoptotic Bax, which could accelerate cytochrome c release from mitochondria to the cytosol, followed by induction of caspase-dependent apoptosis. 15d-PGJ_2_ induced the expression of CDK inhibitor p21 protein in human chondrosarcoma cells, which appears to be involved in the mechanism of inhibition of cell proliferation. These findings suggest that targeted therapy with PPAR*γ* ligands could be a novel strategy against chondrosarcoma.

## 1. INTRODUCTION

Cancers are associated with dysregulation of differentiation and apoptotic cell death. Recent investigations have demonstrated that induction of these cellular events by targeted therapy with ligands of nuclear hormone receptors could be a novel strategy against cancers [1]. Peroxisome proliferator-activated receptor (PPAR)*γ*, a member of the nuclear receptor superfamily, acts as a ligand-activated transcription factor, and is involved in many processes important for homeostasis of cells and tissues, including metabolism, immune and inflammatory controls, cell proliferation and apoptotic cell death [2–6]. Because PPAR*γ* is expressed by many malignant tumors, activation of PPAR*γ* by 15-deoxy-Δ^12,14^-prostaglandin J_2_ (15d-PGJ_2_), the most potent endogenous ligand for PPAR*γ* [7], and the synthetic PPAR*γ* ligands (e.g., rosiglitazone, pioglitazone, troglitazone, and indomethacin) have been regarded as a novel therapeutic approach for certain human malignancies through growth inhibition, induction of apoptosis and terminal differentiation, and inhibition of angiogenesis [8]. This review will outline the inhibitory effects of synthetic and endogenous PPAR*γ* ligands and discuss their potential therapeutic effects on chondrosarcoma.

## 2. CLINICAL FEATURES OF CHONDROSARCOMA

Chondrosarcoma is a malignant tumor of cartilage; the matrix formed by tumor cells is uniformly and entirely chondroid in nature [9]. Human chondrosarcoma is a rare bone tumor, accounting for <10% of primary malignant bone tumors. Chondrosarcoma also arises in pre-existing benign lesions (e.g., osteochondromatosis, enchondromatosis) and is termed “secondary chondrosarcoma.”

Primary (conventional) chondrosarcoma arises centrally in a previously normal bone, and mostly grows slowly through the diaphyseal cortex. Most patients are aged >50 years. Conventional chondrosarcoma is more common in men. The commonest sites are the bones of the pelvis, followed by the femur and the humerus. Recognizable histologic variants are clear cell, mesenchymal, and dedifferentiated chondrosarcomas. On the basis of histologic features (nuclear atypia and cellularity), conventional chondrosarcoma is further subdivided into three grades: I, II, and III [10]. The histologic grade of chondrosarcoma indicates the differentiation status of tumor cells, and is one of the most important factors for prognosis [11]. Progression of a locally aggressive low-grade chondrosarcoma to a metastasizing high-grade chondrosarcoma is associated with loss of cartilaginous phenotype, genomic instability, and aneuploidy [12]. The grading of chondrosarcoma correlates well with clinical behavior, although chondrosarcoma is one of the most difficult malignant tumors of bone to diagnose [13].

Most conventional chondrosarcomas are grade I or II. For low-grade chondrosarcoma, surgical treatment with adequate marginal resection is reported to be associated with better clinical outcomes [14]. Only 5–10% of conventional chondrosarcomas are grade-III lesions, which have definite metastatic potential. The prognosis for high-grade chondrosarcoma is poor, despite adequate surgery, because they are highly resistant to conventional chemotherapy and radiotherapy [15]. These facts, that the differentiation status of chondrosarcoma is predictive of clinical outcomes, suggest the favorable effects of the modification of the differentiation status on clinical behavior. Recent advances in understanding the progression or development of chondrosarcoma have suggested several molecular targets for future development of new adjuvant therapy [16], such as chondrocyte differentiation factors (PTHrP, CTGF) [17, 18], antiapoptotic gene (*Bcl-2*) [19, 20], tumor suppressor gene (*p16*, *p53*) [21, 22], and others (PDGF-*α*, VEGF, *MDR-1*) [23–25].

## 3. OUMS-27, A CHONDROSARCOMA CELL LINE

A cell line derived from chondrosarcoma, particularly from high-grade chondrosarcoma, can provide a useful model for the investigation of cell development and treatment of chondrosarcoma [26–30]. The OUMS-27 cell line has been established from grade III human chondrosarcoma [31]. The cells do not show contact inhibition after reaching confluence, grow rapidly in multiple layers, and express proteoglycan, as well as collagen type I, II, III, IX, and XI after 120 passages, showing stable maintenance of the differentiated chondrocytic properties. The transplantation of OUMS-27 cells into athymic mice resulted in formation of grade II chondrosarcoma at the injection site. There have been many studies on the etiology and treatment of chondrosarcoma using this cell line [32–34].

## 4. EXPRESSION OF PPAR*γ* IN HUMAN CHONDROSARCOMA AND OUMS-27

Subramanian et al. [35] investigated gene expression profiling of ten extraskeletal myxoid chondrosarcomas (EMCs) using 42000 spot cDNA microarrays. Eighty-six genes that distinguished EMC from the other sarcomas were identified by significance analysis of microarrays with 0.25% likelihood of false significance. Of these, PPARG and PPARGC1A, an interacting protein with PPARG and also a coactivator, were highly expressed in EMCs.

In vivo PPAR*γ* protein content was examined in conventional chondrosarcoma specimens from 28 patients undergoing surgery [36]. Immunohistochemical study revealed that human chondrosarcoma cells frequently express PPAR*γ* protein. The positivity (cutoff positivity of 10%) of chondrosarcoma cells were 65.0% in grade I, 83.3% in grade II, and 50.0% in grade III; overall positivity was 67.9% (see Table 1). Expression of PPAR*γ* in OUMS-27 cells at protein and mRNA levels was confirmed by immunocytochemistry and reverse transcriptase-polymerase chain reaction (RT-PCR) analysis, respectively [36]. These data indicated that PPAR*γ* is frequently expressed in primary chondrosarcomas and chondrosarcoma cell line OUMS-27, and led the authors to test the effect of PPAR*γ* activators on cell proliferation and survival of OUMS-27.

## 5. EVIDENCE OF APOPTOTIC CELL DEATH OF OUMS-27 CELLS AFTER TREATMENT BY PPAR*γ* LIGANDS

In our previous report [36], OUMS-27 cells were treated with increasing concentrations of pioglitazone (synthetic PPAR*γ* ligand) and 15d-PGJ2 for up to 48 hours. The results of immunostain for Ki-67 (cell proliferation marker) and colorimetric MTT assay showed that treatment with both pioglitazone and 15d-PGJ_2_ for 24 hours inhibited cell growth and reduced cell viability in a dose-dependent manner, respectively. 15d-PGJ_2_ had more noticeable effects on OUMS-27 cell growth than pioglitazone. It was unclear whether the effects of ligands on OUMS-27 cells were strictly due to PPAR*γ* activation. When cells were treated with 15d-PGJ_2_ doses of ≥5 *μ*g/mL, they showed relatively round shapes and some cells no longer adhered to the dish.

**Figure 1:**
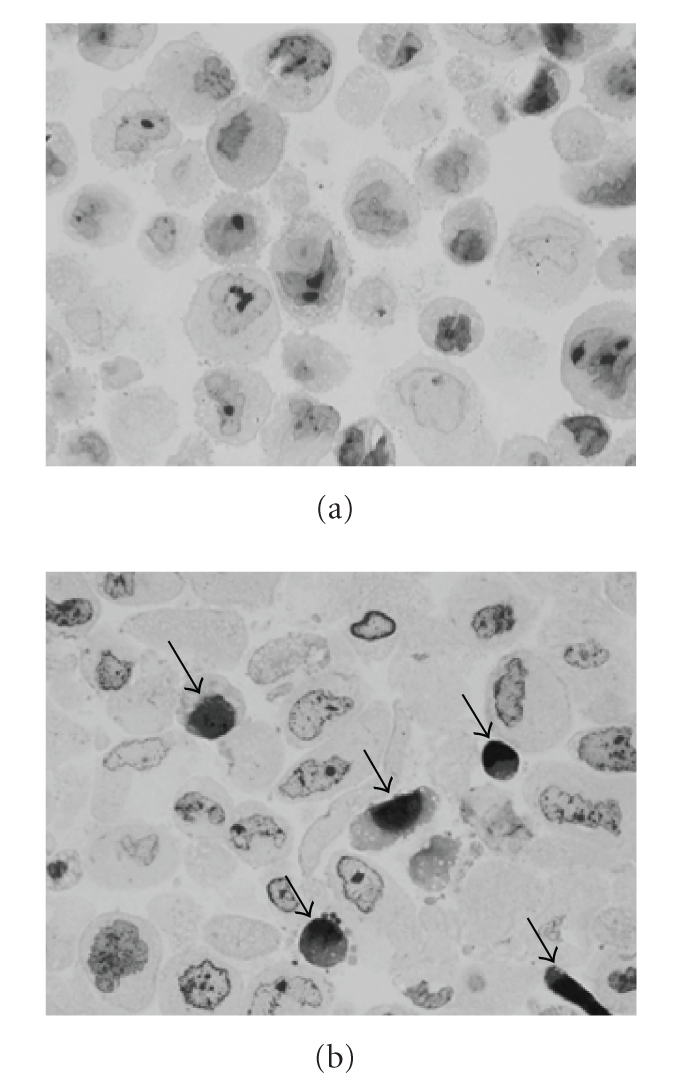
Cell morphology of chondrosarcoma cell line OUMS-27 after incubation with (b) or without (a) 15d-PGJ_2_. Cells were treated with 10 *μ*g/mL of 15d-PGJ_2_ for 8 hours, and the cell pellet embedded in hydrophilic resin. Semithin sections stained by toluidine blue show more apoptotic cells with cell shrinkage and nuclear condensation (arrows) after treatment with 15d-PGJ_2_.

Semithin sectioned, LR White-embedded cells stained by toluidine blue revealed that many OUMS-27 cells treated with 15d-PGJ_2_ show apoptotic appearances with cell shrinkage and nuclear condensation (see Figure 1). DNA fragmentations in OUMS-27 cells treated by 15d-PGJ_2_ (10 *μ*g/mL) for 24 hours were confirmed by DNA ladder formation and TUNEL staining. Transmission electron microscopic study revealed sections of OUMS-27 cells treated with 15d-PGJ_2_ contained many cells consistent with morphological apoptosis with condensed chromatin, many vacuoles in cytoplasm, and membrane budding. Early apoptotic change and the translocation of phosphatidylserine (PS) on the outer leaflet of the cell membrane were demonstrated by FACS analysis. The population of apoptotic cells with PS at the outer membrane of the cells (annexin-V-positive, PI-negative) was ~53.9% and 67.6% at 4 hours and 24 hours after coincubation with 15d-PGJ_2_, respectively.

## 6. MECHANISM OF APOPTOTIC CELL DEATH OF OUMS-27 CELLS BY PPAR*γ* LIGANDS

cDNA microarray analysis was carried out to comprehensively explore the changes in gene expression pattern during OUMS-27 cell growth inhibition and possible cell cycle arrest caused by treatment with 15d-PGJ_2_ [37]. Among the 1081 genes analyzed, 52 genes were upregulated and 81 genes were downregulated significantly in OUMS-27 cells after 8-hour treatment with 15d-PGJ_2_ (10 *μ*g/mL). Microarray analysis is shown in Table 2. Interestingly, the proapoptotic gene Bax was upregulated, and the antiapoptotic gene Bcl-xL was downregulated. The other Bcl-2 members were unchanged. These results were further confirmed by RT-PCR and real-time PCR analysis.

Upregulation of Bax, concurrent with the downregulation of Bcl-xL, can destabilize mitochondria, leading to the release of several mitochondrial intermembrane space proteins such as cytochrome c, AIF, Smac/DIABLO, Endo G, and Omi/HtrA2 into the cytosol, where they are actively involved in apoptotic cell death [38]. This hypothesis is supported by our observations of the release of cytochrome c from mitochondria into the cytosol, and the activation of caspase-3 in 15d-PGJ_2_-treated OUMS-27 cells. Coincubation of cells with the broad-spectrum caspase inhibitor Z-VAD-FMK completely inhibited caspase activity, and prevented the cell death induced by 15d-PGJ_2_. These results indicate that 15d-PGJ_2_ induced apoptosis in OUMS-27 cells through a caspase-dependent signal transduction pathway which, at least in part, was triggered by cytosolic release of cytochrome c [37].

The decreased expression of antiapoptotic Bcl-xL in OUMS-27 treated by 15d-PGJ_2_ led us to examine the expression in the tissue of human chondrosarcoma samples to study the clinical application of differentiation therapy by PPAR*γ* activation. The result of immunohistochemical study demonstrated that Bcl-xL was expressed in all three grades of chondrosarcoma; the expression was strongest in grade III. These results indicated that higher-grade chondrosarcoma cells may be resistant to apoptosis by overexpression of Bcl-xL, and 15d-PGJ_2_ might induce apoptotic cell death by downregulation of Bcl-xL and transient upregulation of Bax [37]. Similar results were reported in renal cell carcinoma cells (786-O and A498 cells) showing the thiazolidinedione (TZD) induction of apoptosis with increased Bax expression and decreased Bcl-2 expression [39].

### 6.1. Genetic and epigenetic alterations in chondrosarcoma

Little is known about the role of genetic or epigenetic alterations in tumor progression from low-malignant chondroblastic to highly malignant anaplastic chondrosarcoma. The appearance of de novo aberrant DNA methylation is the commonest molecular change in the cancer cell, which inactivates many cellular pathways [40]. The most studied change of DNA methylation in neoplasms is the silencing of tumor suppressor genes by deoxy-cytidylatephosphate-deoxy-guanylate (CpG) island promoter hypermethylation, which targets genes and molecules associated in cell differentiation, such as p16(INK4a), BRCA1, and hMLH1 [41–43]. Röpke et al. reported the p16 and E-cadherin promoter methylation in low-grade chondroid compartment of dedifferentiated chondrosarcoma. Van Beerendonk et al. found p16 promoter methylation by methylation-specific PCR in 5 of 30 tumors, but this did not correlate with protein expression, or with loss of heterozygosity (LOH) at 9p21 region, one of the few consistent genetic aberrations found in conventional chondrosarcoma [44]. In OUMS-27, methylation was not detectable in the promoter of p16 gene (unpublished data).

Some reports suggested that p53 mutation and p53 loss of heterozygosity are involved [43, 45]. In OUMS-27, we have previously shown that the p53 gene is mutated [31]. Asp et al. analyzed p16 and p53 in cartilaginous tumor tissues and showed that the p16 gene was found to be partly methylated in 5 high-grade chondrosarcomas and homozygously deleted in 1 chondrosarcoma, whereas the p53 gene revealed an unchanged structure in all 22 chondrosarcoma samples [46].

## 7. INDUCTION OF CELL CYCLE ARREST BY 15d-PGJ_2_ IN OUMS-27

Ligands for PPAR*γ* reportedly induce cell cycle arrest in various cancer cells [39, 47–54]. 15d-PGJ_2_ induces G_1_ arrest and inhibits cell growth of human anaplastic thyroid carcinoma through a p53-independent, but p21- and p27-dependent, manner [55]. Activation of PPAR*γ* by troglitazone inhibited cell growth and induced G_1_ arrest through the increase of cycline-dependent kinase (CDK) inhibitor p27 in several cell lines, including human pancreatic carcinoma cells, gastric cancer cells, and hepatocellular carcinoma cells [56–58]. The effect of troglitazone on the proliferation of cancer cells was inhibited by antisense for p27. Yang et al. also showed TZD decreased the protein levels of proliferating cell nuclear antigen, pRb, cyclin D, and Cdk4, but increased the levels of p21 and p27, in RCC cells [39].

In OUMS-27, 15d-PGJ_2_ induced the expression of the CDK inhibitor p21 protein, and it was increased within 24 hours. Expression of the other CDK inhibitors, p16 and p27 proteins, were detected at time zero, and were not significantly influenced by 15d-PGJ_2_ treatment [37]. 15d-PGJ_2_-induced p21 may exert cell cycle arrest in a p53-independent manner.

Whether 15d-PGJ_2_ induces p21 expression in OUMS-27 cells through a PPAR*γ*-dependent or -independent pathway is unclear. It is possible that p21 expression is directly regulated by PPAR*γ* activation because p21 gene contains a potentially conserved consensus PPAR*γ* response element in the promoter region [59]. Copland et al. reported [60] that RS5444, a novel high-affinity PPAR*γ* agonist, inhibits anaplastic thyroid cartinoma (ATC) tumor growth and angiogenesis in mice. In DRO cells derived from ATC tumor, they demonstrated that upregulation of p21 by RS5444 is PPAR*γ* dependent, and might be the major mechanism by which RS5444 inhibits DRO cell proliferation. Han et al. demonstrated the link of PPAR*γ* activation and p21 signaling to cell growth inhibition in human lung cell cartinoma cells using p21 antisense oligonucleotides [61]. They also indicated the induction of p21 expression by PPAR*γ* ligands might be mediated through increased Sp-1 and NF-interleukin 6 (IL6) CAAT/enhancer binding protein (C/EBP)-dependent transcriptional activation.

## 8. CLINICAL APPLICATION OF PPAR*γ* AGONIST FOR CHONDROSARCOMA SUPPRESSION

Accumulating evidence suggests that PPAR*γ* activators might have clinical therapeutic benefit in the treatment of cancers. Although initial clinical trials with troglitazone reported promising results in liposarcomas [62] and prostate cancers [63], recent studies failed to show the expected therapeutic values of rosiglitazone in liposarcomas [64] and early-stage breast cancers [65], and troglitazone in chemotherapy-resistant metastatic colorectal cancers [66]. However, a single study of a phase-I clinical trial of LY293111 in patients with advanced solid tumors reported a potential efficacy of PPAR*γ* agonist for chondrosarcoma [67]. LY293111 is an orally stable leukotriene B_4_ (LTB_4_) receptor antagonist, as well as a PPAR*γ* agonist, as demonstrated by activity in the rat ZDF diabetes model, and the induction of adipocyte differentiation. One patient with progressive chondrosarcoma had stable disease lasting ~336 days of LY293111 administration at the dose of 200 mg bd.

## 9. FUTURE DIRECTION

In chondrosarcoma, whether the cell death and growth inhibitory effects induced by 15d-PGJ_2_ are PPAR*γ*-dependent or -independent is unknown. As 15d-PGJ_2_ at high doses is toxic for most of cell types independent of PPAR*γ* activation, we examined the effects of the caspase inhibitor Z-VAD-FMK, and the PPAR*γ* antagonist GW9662, on caspase-3 activation and cell viability of OUMS-27 cells treated by 15d-PGJ_2_ [37]. 15d-PGJ_2_ alone clearly increased cell death; the addition of GW9662 partially inhibited cell death. Cell death was inhibited almost to control level when Z-VAD-FMK was added to 15d-PGJ_2_. The activity of caspase-3 was attenuated, though not completely, by stimulation of 15d-PGJ_2_ together with GW9662. These data indicate that the greater proapoptotic effects of 15d-PGJ_2_ on chondrosarcoma cells may result from the cumulative effects of PPAR*γ*-dependent and -independent pathways. Detailed analysis of the effects of ligands on cells transfected with PPAR*γ* siRNA should provide important clues to understanding this phenomenon. Whether endogenous or synthetic PPAR*γ* ligands can also induce tumor cell death in an experimentally transplanted chondrosarcoma model remains to be examined before human trial.

## Figures and Tables

**Table 1:** Summary of immunohistochemical study for PPAR*γ* in human chondrosarcoma tissues.

Positive cell	Pathological grade of chondrosarcoma (%)		
ratios (%)	I (*n* = 20)	II (*n* = 6)	III (*n* = 2)
<10	35	17	50
10–40	40	33	50
>40	25	50	0

**Table 2:** Changes in apoptosis-related gene expression in OUMS-27 cells after treatment with 15d-PGJ_2_.

Gene name	Fold
Clusterin	+3.7
Defender against cell death 1	+3.8
Tumor necrosis factor receptor 1	+3.2
State-induced state inhibitor 2	+2.5
Heat shock protein 60	+2.4
V-akt murine thymoma viral oncogene homolog 1	+2.3
Apoptosis regulator bax	+2.3
Interferon-induced RNA-dependent protein kinase	+2.1
Apoptosis regulator bcl-xl	−2.2
Calpain, small subunit 1	−2.3
Heat shock 70 KD protein 1	−2.4
Endothelin 2	−2.4
Insulin-like growth factor 1 receptor	−2.5
Death-associated protein 6	−2.8
